# A multifaceted training approach for neurosurgery registrars incorporating cadaver-based simulation

**DOI:** 10.4102/jcmsa.v4i1.287

**Published:** 2026-04-22

**Authors:** Mohammed Z. Ebrahim, Rhoda Meyer, Elize Archer

**Affiliations:** 1 Department of Neurosurgery, Faculty of Medicine and Health Sciences, Stellenbosch University, Cape Town, South Africa; 2 Department of Health Professions Education, Faculty of Medicine and Health Sciences, Stellenbosch University, Cape Town, South Africa

**Keywords:** registrar perceptions, neurosurgery training, skills, cadaver-based simulation, entrustable professional activities, competency-based education

## Abstract

**Background:**

Neurosurgical training has historically been grounded in an apprenticeship model. This approach to developing competence relies on authentic learning opportunities presenting themselves in the operating theatre. Recently, aspects such as medical litigation, increasing registrar numbers, reduction in mandatory work hours and the need to prioritise service delivery over training opportunities have led to the incorporation of cadaver-based simulation training to increase surgical exposure. Our study aimed to explore how a structured training package in anterior cervical discectomy and fusion influences a group of neurosurgery registrars’ perception of their skills development.

**Methods:**

The training package consisted of an interactive lecture, a video and two cadaver-based simulation training sessions. The study population included seven registrars. Data was generated through a questionnaire completed at recruitment and semi-structured interviews conducted after exposure to the training package. Demographic information was captured and summarised, and the interviews were analysed using thematic analysis.

**Results:**

Structured training in a basic neurosurgical technique was found to be valuable and preferred to fragmented and opportunistic learning opportunities. It promoted confidence in procedural principles and theatre setup and provided invaluable opportunities for learning. Opportunities to practise basic techniques made the theatre environment less hostile and promoted learning in the workplace.

**Conclusion:**

Structured training packages incorporating cadaver-based simulation are a potentially important component of skills development in neurosurgical training.

**Contribution:**

Such learning resources are based on neurosurgical core competencies and can form part of a shift towards a more comprehensive competency-based training programme.

## Introduction

Neurosurgical training, as with most surgical disciplines, is grounded in an apprenticeship model.^1^ This approach to developing surgical competence relies on learning opportunities presenting themselves in the operating theatre.^2^ However, the training arena has changed over the last few decades. There has been an increase in the number of registrars being trained per unit and a global shift towards the reduction in mandatory work hours.^3^ It has also become increasingly challenging to mitigate the risk of potential medical litigation and ensure the availability of adequate training opportunities while keeping up with the demands of service delivery.^4^ There is, however, a need to train more neurosurgeons to meet the growing demands in the healthcare system, especially in under-resourced settings.^5^ In the South African setting, neurosurgeons must be trained in a wide range of skills in order to practise safely and independently. To facilitate this, adequate exposure to opportunities for developing these skills is essential.

Neurosurgery comprises a variety of surgical approaches and techniques of variable complexity and risk that can only be mastered through practice.^6^ Expert performance of an acquired skill can be linked to active engagement in deliberate practice.^7^ A training approach based on deliberate practice is grounded in providing consistent educational intervention, promoting progressive skills development and maintenance of the skills acquired.^8^ In developing surgical competence, more opportunities for deliberate practice need to be integrated into training, and one way to achieve this is through simulation training.^9^ Cadaver-based simulation training provides an environment to acquire familiarity with neurosurgical techniques, void of all the real-world risks and stressors.^10^ It promotes the development of surgical dexterity and familiarity with the relevant surgically orientated anatomy.^6^

The introduction of structured training packages based on core neurosurgical competencies has been considered in our unit. These training packages incorporate theoretical knowledge with cadaver-based simulation training to create more opportunities for practice and skill acquisition. These are not merely ad hoc simulation workshops but rather training opportunities integrated into the training programme, which could later be structured into an ongoing workplace-based assessment framework. The efficiency of a structured training programme over one that relies on ad hoc learning opportunities has been shown to improve the quality of surgical education.^11^ The Colleges of Medicine of South Africa has adopted a Competency-based Medical Education framework (CBME) involving workplace-based assessment and entrustable professional activities (EPA). The EPA involved in the training package used for this study was an anterior cervical discectomy and fusion (ACDF).

There is a relative paucity in the literature on the perceptions of registrars on simulation as a teaching method, and the effect that training packages have on their skills development.^9^ Furthermore, little is known about their perceptions of simulation training in a cadaver lab setting. This study thus set out to explore how a structured training package, which included cadaver-based simulation training, influenced neurosurgery registrar’s perceptions of their skills development in the ACDF procedure.

## Research methods and design

### The training package

**FIGURE 1:**
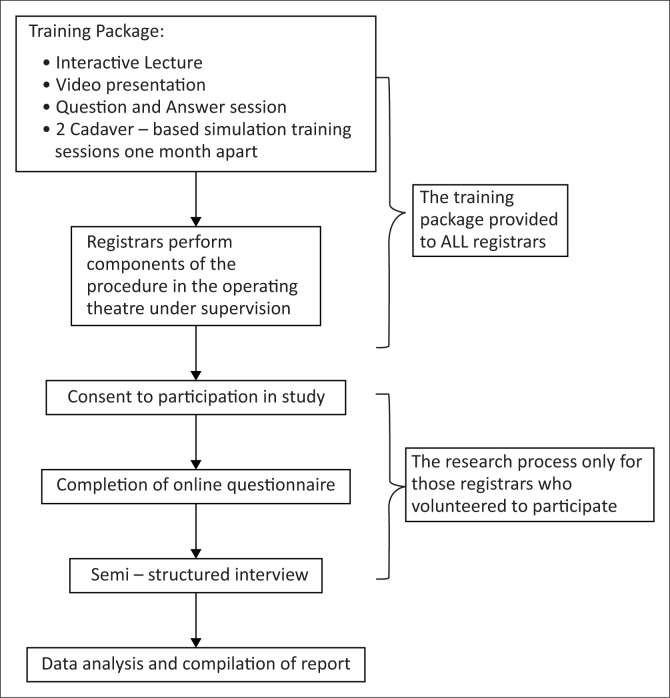
The research process.

The training package consisted of a 20-min interactive PowerPoint lecture on the fundamental principles of performing an ACDF. A video demonstrating the basic procedural steps was then presented together with a question-and-answer (see Figure 1) session and a 2-h cadaver-based simulation training session. This simulation session allowed the registrars to perform the procedure on cadavers under supervision, focussing on the key components and guided by the pre-determined core competencies. A junior registrar was paired with a senior registrar at each cadaver. A second simulation session was held 1 month thereafter. This session provided the opportunity to revisit any areas of uncertainty and practice the critical steps again. Following this, the registrars were given 3 months to perform components of this procedure in the theatre under supervision. It is important to note that the operations were not specially scheduled for the purpose of the study, but rather formed part of the registrar’s usual clinical duties within the department. Therefore, no patient was placed at any additional risk as the regular departmental procedures for the theatre list and registrar allocation to each list were applied.

### Study design and setting

This qualitative study utilised a descriptive approach. The study took place within a neurosurgical department at a tertiary hospital in South Africa.

### Sampling and participant recruitment

The total population of seven registrars in the Department of Neurosurgery at the time were approached to participate in this study, and no sampling was performed. Recruitment was done via email, and informed consent was obtained.

### Data generation

Data was generated through an online questionnaire and semi-structured interviews. The registrars who provided consent to participate were asked to complete a questionnaire related to aspects of previous experience in neurosurgery. This questionnaire aimed to gather descriptive data on the registrar population in the department. Semi-structured interviews were conducted with each participant individually. Attempts to minimise the power dynamics at play during the interview were made by establishing trust through assuring confidentiality and reiterating that the process was aimed at refining training and in no way aimed at assessment. The interviews were audio–recorded and transcribed using the Otter.ai transcription tool. The researcher and the study supervisors checked these transcriptions for accuracy. Member checking was utilised to ensure trustworthiness.

### Data analysis

#### The questionnaire

Through the questionnaire, demographic and background information were captured and summarised.

#### The interviews

The data analysis of the individual interviews followed an iterative process and was guided by Braun and Clarke’s six steps of thematic analysis.^12^ The process began by reading and re-reading the transcripts to identify possible patterns related to the research question. Sections of text were then labelled to create codes, which were grouped to form categories. Similar categories were combined, which then culminated in a set of interrelated themes. These themes are presented and explored below.

### Ethical considerations

The study was reviewed and approved by the Health Research Ethics Committee and the institution (ethics reference number: S23/07/154). Participants provided written informed consent. All interview transcripts were de-identified. All collected data, including voice recordings and transcripts, were kept in a password-protected electronic folder in the possession of the primary researcher.

## Results

### Findings from the questionnaire

Five registrars volunteered to participate in this study. All participants were at least 5 years beyond graduation from medical school and had at least 2 years of neurosurgical experience.

Experience in spinal surgery and specifically Anterior cervical discectomy and fusion (ACDF) varied and did not correlate to their Year of study in neurosurgery training programme (YOS). The junior registrars, those in training years 1 to 3, expressed a lack of appropriate exposure to basic spinal procedures to promote confidence in their skills development. Their exposure in the spinal rotations was largely to procedures of a complexity not suitable for effective junior registrar training. The exposure among senior registrars in training years 4 to 5 demonstrated great variation in exposure and perceived competence in certain aspects of spinal surgery.

The information that was gathered with the questionnaire assisted in phrasing the prompts for the interviews, and during the interviews, these aspects were explored in more depth.

### Findings from the interviews

#### Influence of the simulated learning environment on skill acquisition

This theme relates to the potential of the simulated learning environment to influence skills development. It includes aspects, such as opportunities to practise in a low-risk environment, ease of access to cadavers and the value of learning in simulation in preparation for the workplace.

Registrars found value in opportunities to practise in the low-risk environment of the cadaver lab. It allowed them to familiarise themselves with surgically oriented anatomy without the complications accompanying inexperience in exposing the relevant anatomical structures:

‘[*I*]nteract with the bone, see what the bony anatomy and its relations are in a setting where you are not worried about catastrophic complication. You can see how close these structures are, and you can get a feel for it …’ (Registrar 5, YOS 5)

Learning in this environment also allowed for trial and error during the process of developing familiarity with complex surgical skills. Participants felt more at ease because there is no risk to a patient:

‘I find that whenever I am able to do a procedure, especially in the skills lab on cadavers, every time I go there, we will try something new.’ (Registrar 5, YOS 5)

Registrars felt that the cadaver lab enabled an effective environment for simulation training in terms of approximating a real-life theatre setting:

‘It is great to go and practice on cadavers, obviously, because that is not a procedure we get a lot of practice on, on actual patients. Being able to practice them on cadavers was a valuable resource.’ (Registrar 3, YOS 2)

Registrars suggested that practising surgical techniques and exploring anatomical relationships in a guided manner better equips them to have more meaningful learning experiences in theatre:

‘So the last time you were there, we looked at actually going all the way lateral and actually opening the transverse foramen, opening the vert and seeing where it ran. Today that actually came in very useful because I had to go far lateral …’ (Registrar 5, YOS 5)

In some cases, participants referred to how the cadaver-based simulation complemented other aspects of the training package. For example, it was suggested that an intraoperative experience, which may be challenging without prior exposure to the anatomical relationships, was dealt with effectively, and the valuable real-world learning opportunity was taken full advantage of without compromising patient safety:

‘And it is not about being bullish; it is more about knowing where I’m safe and when I am at a point where I need some more experience to take over, and today I felt that it was safe, the patient was doing well, so it was nice to have it.’ (Registrar 1, YOS 3)

### Value of structured learning opportunities

This theme describes registrars’ perceived value of structured learning opportunities and their preference over a fragmented format. Although at times, it includes aspects of the full training package, it highlights how the cadaver-based simulation contributed to improving the registrar’s overall learning experience.

Registrars found the structure and content of the training package to have significant educational value, suggesting that similar packages for other basic neurosurgical techniques are necessary:

‘I am relieved to receive training, and specifically with ACDF training, it is nice to finally receive information where it is systematic, and it is not just picking it up here and there and finding it out on the go. I was pleased to have that in one place.’ (Registrar 1, YOS 3)

The inclusion of real-life examples and cases appeared to have assisted in cultivating the cognitive expertise necessary to support the development of skills:

‘So to have actual examples of anatomy and trajectories and reasoning and explanations and pearls and pitfalls was phenomenal.’ (Registrar 5, YOS 5)

The expert opinion provided during the training package creates the contextual framework for learning and skills development to occur:

‘It is one thing to go and read the procedure, read the indications in the textbook, but if you don’t have the clinical context or the experience, it often helps to have someone who has done them, tell you.’ (Registrar 1, YOS 3)

Developing the skills to perform a procedure safely is facilitated by opportunities to practise in a cadaver lab and by the discussions around the key aspects of the procedure:

‘I think that any exposure or any conversation around these procedures always enlightens me in various ways. Any conversation, any discourse or talking about these procedures, you always manage to learn something or even just reiterate something that, that you never said out loud.’ (Registrar 1, YOS 3)

### Mentorship and collaborative learning

This theme relates to acquiring skills through mentors and more experienced colleagues, reflecting participants’ experiences of the training package as a whole.

It was clear that registrars often rely on learning through observation and insights of peers and mentors:

‘If you want to do these operations, you need to come watch the masters at work.’ (Registrar 5, YOS 5)

Registrars also suggested that observing a variety of surgeons allows one to learn different skills. In addition to consultant input and feedback, registrars valued interactions with other, often more senior, registrars. This peer-to-peer learning is often thought of as a good precursor to teaching and learning interactions with their consultants:

‘I believe that anyone is able to train; it is not just the consultants; it is the senior guys as well.’ (Registrar 1, YOS 3)

### Bridging the gap between simulation and reality

This theme relates to the development of skills and their application in the real-world context, specifically after engaging in cadaver-based simulation.

Registrars acknowledge that there is a difference between practising a procedure in a cadaver lab versus observing it being performed or performing it independently in real practice:

‘[*R*]egardless of how many ACDFs I have seen and assisted, it is a completely different ballgame to have to do it yourself …’ (Registrar 3, YOS 2)

The transition from the demonstration of a procedure to the performance of that procedure independently can be a more difficult step to take:

‘I realised that there is such a discrepancy between the two in the sense of my experience currently and the next step to operating alone. I can recite the steps of an ACDF verbatim, but when you put the instruments in my hands, it is a completely different thing, and I am suddenly out of my depth. There is that bridge that needs to be crossed.’ (Registrar 3, YOS 2)

‘So it is just extra exposure that is helping to bridge the gap …’ (Registrar 5, YOS 5)

Opportunities to practise and gain experience in the nuances of a procedure equips one to adapt effectively to the unpredictable workplace environment:

‘Your ability to maybe kind of work under other kinds of situations is only seen by seeing more, so you can kind of troubleshoot.’ (Registrar 1, YOS 3)

## Discussion

Neurosurgery is a high-risk speciality with minimal margin for error.^13^ In this study, the responses to the questionnaire illustrated the disparity in exposure to surgical procedures among junior and senior registrars. It also illustrated that senior registrars did not receive similar surgical exposure in certain procedures. This needs to be an area of focus in order to standardise exposure and training. In an unstructured teaching system, the opportunities for exposure and learning are unequal. This is echoed in other studies where trainees have reported heterogeneity in practical training within academic centres and between different centres, resulting in varying degrees of competence among graduates^14,15^

The learning environment has an influence on skill acquisition. The use of cadaver-based simulation allows learning to occur in a low-risk environment. This may allow for a more efficient acquisition of basic surgical skills,^10^ by promoting an environment for skill acquisition away from the high-stakes, stressful theatre environment. Registrars are able to gain familiarity with the components of a procedure before having to perform the same steps in theatre on a real patient. The overall perception of the registrars was that learning can occur in both the theatre setting (workplace) and the skills lab (simulation), but that learning in simulation formed a beneficial precursor to workplace-based training and assessment.

The registrars reported several benefits to structured learning opportunities. This perception is supported in the literature, where structured training programmes are shown to be useful in optimising the quality of surgical education by promoting skills development and maintenance of the skills acquired through deliberate practice.^1,8,11^ Simulation training packages, such as the one described in this study, lend themselves to this structured approach to neurosurgical training. It creates a platform for training in the foundational elements of an otherwise seemingly overwhelming skill set that registrars need to acquire on an ad hoc basis.

Registrars typically acquire competence in procedural skills through observation and exposure.^16,17^ Clinical educators therefore need to acknowledge that they are role models and be acutely aware of the influence they have directly and indirectly on the skills development of registrars. Effective role models should display clinical competence, a degree of teaching ability and personal qualities worthy of emulation.^18^ Learning through role-modelling not only involves skills acquisition but also extends to the application of the foundational knowledge and attitudes towards the treatment of patients with certain pathologies. In this study, the registrars found value in observing seniors performing surgery. In the realm of skills development, senior clinicians need to embrace the role of teacher because all of the actions and attitudes of an expert are potentially key learning points for a novice.

However, peer supervision in the operating theatre often occurs more frequently than supervision by consultants or faculty members.^15^ In our study, the training package enabled peer learning as junior registrars were paired with senior registrars at the dissection tables. This allowed immediate feedback from a senior colleague and input from the circulating consultant at the session, which was valuable for the registrars’ learning. However, deliberate practice should always be paired with feedback to avoid reinforcing incorrect techniques. This is consistent with what McGaghie et al. argue that feedback is an important component of the principles of deliberate practice as it promotes a learning environment that monitors progress and error correction in the skill acquisition process.^8^

The success of this training package lies in the integration of interactive teaching and learning with simulation, providing a framework to engage with experts and mentors on the pearls and pitfalls of a procedure in a simulated environment. The perceived value is enhanced if authentic practice within the operating room is possible soon after the simulation training. In future, these packages should include spinal and cranial approaches, which lend themselves to cadaver-based simulation training. This should form part of a skills training curriculum that complements the programme’s existing theoretical component. These principles can also be applied to other specialities.

As postgraduate medical education moves more towards workplace-based assessments, it is important that clinical educators are trained to facilitate these training packages effectively through tailored faculty development initiatives. A potential area of focus for future research can include the effect of the incorporation of structured training packages in skill development on registrar competence and confidence.

### Limitations

The findings from this study only represent the experiences and perceptions of five out of the seven registrars at our neurosurgical unit. Additionally, findings are not based on observation or assessment of knowledge or performance of the required skills, but on individual registrar perceptions.

## Conclusion

This study explored how a structured training package in ACDF influences the neurosurgery registrar’s perception of their skills development. The study showed that the training package was perceived to be of value to our registrars. It enhanced their learning and promoted confidence in procedural skills, facilitating feedback and skills development in the operating theatre. This study has found that within our neurosurgery department, a move towards introducing structured training packages in core neurosurgical competencies may promote a more comprehensive training programme.
